# MEDICATIONS ASSOCIATED WITH GERIATRIC SYNDROMES AND HOSPITALIZATION RISK IN HOME HEALTHCARE PATIENTS

**DOI:** 10.1093/geroni/igac059.1448

**Published:** 2022-12-20

**Authors:** Jinjiao Wang, Jenny Shen, Fang Yu, Yeates Conwell, Yue Li, Thomas Caprio, Avantika Shah, Sandra Simmons

**Affiliations:** University of Rochester School of Nursing, Rochester, New York, United States; University of Rochester Medical Center, Rochester, New York, United States; Arizona State University, Phoenix, Arizona, United States; University of Rochester Medical Center, Rochester, New York, United States; University of Rochester, Rochester, New York, United States; University of Rochester, Rochester, New York, United States; Johns Hopkins University, Baltimore, Maryland, United States; Vanderbilt University Medical Center, Nashville, Tennessee, United States

## Abstract

Polypharmacy is common in the home health care (HHC) setting. This study examined the prevalence, predictors, and impact of medications associated with geriatric syndromes (MAGS) use on subsequent hospitalization in HHC patients. Data from the HHC electronic medical records, the Outcome and Assessment Information Set (OASIS), and Medicare HHC claims of 6,882 adults ≥ 65 years old receiving HHC from a large, non-profit HH agency in New York State in CY 2019 were used. MAGS use was identified from active medications reconciled during HHC visits and defined as total count and in quartiles. Hospitalization during the HHC episode was operationalized as a time-to-event variable. Regression analyses were conducted to identify predictors of MAGS use, and survival analyses were conducted to examine the association between MAGS use and hospitalization. Nearly all (98%) of the HHC patients used at least one MAGS and 41% of all active medications were MAGS. A higher MAGS count was found among HHC patients who were non-white, community-referred, with more comorbidities, depressive symptoms, and functional limitations. A higher MAGS count was also related to increased risk for hospitalization. Moreover, higher quartiles of MAGS use combined with having ≥10 diagnoses predicted a 2.5-fold increase in hospitalization risk, relative to the lowest quartile of MAGS use and having < 10 diagnoses. In conclusion, over 40% of medications taken by HHC patients are MAGS, which, along with multimorbidity, increased hospitalization risk. HHC clinicians should identify MAGS use to inform deprescribing discussion with patients and their prescribers.

